# Effects of sound intensity on temporal properties of inhibition in the pallid bat auditory cortex

**DOI:** 10.3389/fphys.2013.00129

**Published:** 2013-06-03

**Authors:** Khaleel A. Razak

**Affiliations:** Department of Psychology, Graduate Neuroscience Program, University of CaliforniaRiverside, CA, USA

**Keywords:** pallid bat, echolocation, FM sweeps, sideband inhibition, spectrotemporal

## Abstract

Auditory neurons in bats that use frequency modulated (FM) sweeps for echolocation are selective for the behaviorally-relevant rates and direction of frequency change. Such selectivity arises through spectrotemporal interactions between excitatory and inhibitory components of the receptive field. In the pallid bat auditory system, the relationship between FM sweep direction/rate selectivity and spectral and temporal properties of sideband inhibition have been characterized. Of note is the temporal asymmetry in sideband inhibition, with low-frequency inhibition (LFI) exhibiting faster arrival times compared to high-frequency inhibition (HFI). Using the two-tone inhibition over time (TTI) stimulus paradigm, this study investigated the interactions between two sound parameters in shaping sideband inhibition: intensity and time. Specifically, the impact of changing relative intensities of the excitatory and inhibitory tones on arrival time of inhibition was studied. Using this stimulation paradigm, single unit data from the auditory cortex of pentobarbital-anesthetized cortex show that the threshold for LFI is on average ~8 dB lower than HFI. For equal intensity tones near threshold, LFI is stronger than HFI. When the inhibitory tone intensity is increased further from threshold, the strength asymmetry decreased. The temporal asymmetry in LFI *vs*. HFI arrival time is strongest when the excitatory and inhibitory tones are of equal intensities or if excitatory tone is louder. As inhibitory tone intensity is increased, temporal asymmetry decreased suggesting that the relative magnitude of excitatory and inhibitory inputs shape arrival time of inhibition and FM sweep rate and direction selectivity. Given that most FM bats use downward sweeps as echolocation calls, a similar asymmetry in threshold and strength of LFI *vs*. HFI may be a general adaptation to enhance direction selectivity while maintaining sweep-rate selective responses to downward sweeps.

## Introduction

Bats of the suborder, microchiroptera, can be broadly classified as constant frequency-frequency modulation (CF-FM) or frequency modulation (FM) bats based on their echolocation calls (Jones and Teeling, 2006). The diversity of echolocation call structure within these broad classes provides the opportunity to explore the evolution of diverse spectral and temporal neural processing strategies using behaviorally-relevant sounds. Studies of auditory neurons in CF-FM and FM bats reveal selective responses to features present in the species-specific echolocation calls (Suga et al., 1987; Suga, 1989; Dear et al., 1993; Wenstrup et al., 2012). In terms of FM sweeps, neural selectivity for the rate and direction of change in frequency have been well characterized (Casseday and Covey, 1992; Gordon and O'Neill, 1998; O'Neill and Brimijoin, 2002; Gittelman et al., 2009; Fuzessery et al., 2011; Washington and Kanwal, 2012).

Beginning with studies by Suga (1965), one conceptual framework to study mechanisms underlying FM sweep rate and direction selectivity is based on asymmetries in sideband inhibition. Auditory neurons, like those in the visual and somatosensory systems, exhibit excitatory and inhibitory components in the receptive field (Calford and Semple, 1995; Brosch and Schreiner, 1997; Gordon and O'Neill, 1998; Sutter et al., 1999; Faure et al., 2003; Wehr and Zador, 2003; Razak and Fuzessery, 2006; Wu et al., 2008; Sadagopan and Wang, 2010; Kuo and Wu, 2012). The inhibitory sideband refers to frequencies of sounds that do not elicit an excitatory response when presented alone, but can suppress spontaneous or excitatory sound-evoked responses. Such inhibitory frequencies are present below the low-frequency edge (low-frequency inhibition, LFI) and/or above the high-frequency edge (high-frequency inhibition, HFI) of the excitatory tuning curve (henceforth, “tuning curve”). In its simplest form, the asymmetry hypothesis suggests that absent inhibition on one side of the tuning curve will cause a neuron to be sweep direction selective.

More recent studies have provided refinement to this hypothesis as well as identified additional mechanisms such as facilitation and duration tuning to explain FM sweep selectivity across different levels of the auditory system and across species (Gordon and O'Neill, 1998; Fuzessery et al., 2006; Razak and Fuzessery, 2006, 2008; Gittelman et al., 2009; Sadagopan and Wang, 2010; Trujillo et al., 2013). It is now established that it is not simply the presence or absence of inhibition on either side of the excitatory tuning curve that shapes FM sweep direction/rate selectivity. More complex interactions between the relative bandwidth, timing and strength of inhibitory and excitatory inputs are involved.

The pallid bat (*Antrozous pallidus*) has served as a useful model in these investigations because of a high percentage of neurons selective for FM sweeps used in echolocation (Fuzessery et al., 2006, 2011; Razak and Fuzessery, 2006). The pallid bat echolocates using downward FM sweeps [60→30 kHz, 2–5 ms duration (Barber et al., 2003)]. Between 65–75% of neurons tuned between 25–70 kHz in the inferior colliculus (IC) and auditory cortex are selective for the downward direction and the range of sweep rates present in the echolocation call (Fuzessery, 1994; Razak and Fuzessery, 2002). The two-tone stimulation paradigm has been used to study underlying mechanisms. In this method, two tones are presented with different delays to characterize spectral and temporal interactions within the receptive field in neurons with known FM rate and direction selectivity (Gordon and O'Neill, 1998; Fuzessery et al., 2006; Razak and Fuzessery, 2008, 2006). These studies showed that most neurons have sideband inhibition on both sides of the tuning curve, but show temporal asymmetries such that HFI arrives later than excitation and LFI arrives earlier than excitation. Upward sweeps will first pass through the LFI which will arrive at the neuron before and during excitation to squelch responses. Downward sweeps with fast sweep rates reach the excitatory frequencies before the delayed HFI arrives and elicit a neural response. For slow downward sweeps, the delayed HFI has sufficient time to arrive at the neuron with or before the excitation and reduces responses. Thus, early LFI and delayed HFI shape direction and rate selectivity for downward FM sweeps, respectively. Removal of LFI from the sweep or reducing inhibition with GABAa receptor antagonists reduces direction selectivity (Razak and Fuzessery, 2009; Williams and Fuzessery, 2011). Likewise, removing HFI from the sweep or iontophoresis of GABAa receptor antagonists reduces rate selectivity.

In previous studies, sideband inhibition was determined using two tones that were presented at the same intensity. Recent studies suggest that the temporal interactions between excitatory and inhibitory inputs are modulated by the relative strength of inhibition and excitation (Wu et al., 2006; Gittelman et al., 2009; Razak, 2012). Temporal asymmetries in the pallid bat cortex may therefore arise from differences in the strength of HFI and LFI. The main goal of the present study was to characterize sideband inhibition in the auditory cortex by varying both relative intensity and time delays between tones in the two-tone paradigm. This paradigm allows a quantification of the intensity-arrival time relationship between excitatory and inhibitory frequencies. The data show that: (1) the threshold of LFI is lower than the threshold of HFI and, (2) if the relative intensity of the inhibitory tone is increased, the arrival time decreases for both HFI and LFI, and temporal asymmetry decreases.

## Materials and methods

Pallid bats were netted in Arizona, California and Texas and housed in a 11 × 14 ft room. The bats were able to fly in this room and were provided crickets/mealworms and water *ad libitum*. The room was maintained on a reversed 12:12 light cycle. All procedures followed the animal welfare guidelines required by the National Institutes of Health and the Institutional Animal Care and Use Committee.

### Surgical procedures

Recordings were obtained from the right auditory cortex of bats (both males and females, *n* = 9 bats) anesthetized with isoflurane or methoxyflurane inhalation, followed by an i.p. injection of urethane (0.7 mg/g) or pentobarbital sodium (30 μg/g). A previous study comparing urethane and barbiturate anesthetics showed no differences in FM sweep selectivity or arrival time/bandwidth of sideband inhibition (Razak and Fuzessery, 2009) Therefore, the data obtained using the different anesthetics were combined here. To expose the auditory cortex, the head was held in a bite bar, a midline incision was made in the scalp, and the muscles over the dorsal surface of the skull were reflected to the sides. The front of the skull was scraped clean and a layer of glass microbeads applied, followed by a layer of dental cement. The bat was then placed in a Plexiglas holder. A cylindrical aluminum head pin was inserted through a cross-bar over the bat's head and cemented to the previously prepared region of the skull. This pin served to hold the head secure during the recording session. The cross-bar holding the head pin was secured behind the bat, leaving no interference between the speaker and the ear. The location of A1 was determined relative to the rostrocaudal extent of the midsagittal sinus, the distance laterally from the midsagittal sinus, and the location of a prominent lateral blood vessel that lies parallel to the midsagittal sinus. The size of the exposure was usually ~2 mm^2^. Exposed muscle was covered with petroleum jelly, and exposed brain surface was covered with silicone oil to prevent desiccation.

### Recording procedures

Experiments were conducted in a warm (~80°F), sound-proof chamber lined with anechoic foam (Gretch-Ken Industries, Oregon). Bats were kept anesthetized throughout the course of the experiments with additional urethane or pentobarbital sodium (one-third of pre-surgical dose) injections. Acoustic stimulation and data acquisition were driven by custom software and Microstar DSP board based hardware. Programmable attenuators (PA5, Tucker-Davis Technologies, Florida) allowed control of sound intensities before amplification by an integrated amplifier (Yamaha AX430). Stimuli were delivered either using an LCY-K100 ribbon tweeter (Madisound, Wisconsin) placed 8 in from the left ear at 45° to the long-axis of the bat's body or presented as contralateral ear closed-field stimuli through the ribbon tweeters fitted with funnels. Each neuron reported in this study was tested with one or the other method (free-field or closed-field). Preliminary data from neurons in which the closed-field and free-field data were compared indicated that the minimum thresholds (MTs) were ~5 dB higher for the free-field presentation. Because all neurons in this study were tested with excitatory tones presented at 10–20 dB above threshold regardless of the presentation method used, it is unlikely that the results were due to the presentation method. Most FM sweep selective neurons tuned between 30–60 kHz in the pallid bat auditory cortex are also binaurally insensitive (EO/O type neurons) when tested with interaural intensity differences (Razak and Fuzessery, 2002). Therefore, IID sensitivity is unlikely to create differences between free-field and closed-field data. The frequency response curve of the delivery systems, measured with a 1/4-in microphone (Bruel and Kjaer, Denmark), was flat within ±5 dB for frequencies from 6–50 kHz. The roll-off from 50–80 kHz was gradual at a rate ~20 dB/octave.

Data shown are from extracellular single-unit recordings identified based on window discriminator threshold-crossing and consistency of action potential amplitude and waveform displayed on an oscilloscope. Recordings were obtained using glass electrodes (1M NaCl, 2–10 MΩ impedance) at depths between 200 and 600 μm. Penetrations were made orthogonal to the surface of the cortex. Action potentials were amplified by a Dagan extracellular preamplifier (2400A) and a spike signal enhancer (FHC, Maine) and band-pass filtered (0.3–3 kHz, Krohn-Hite, MA). Waveforms and peri-stimulus time histograms were stored. Responses were quantified as the total number (20 stimulus repetitions, 1 Hz repetition rate) of action potentials occurring within 200 ms of stimulus onset. Adjustments for spontaneous activity were not necessary because there was no spontaneous activity in these recordings.

The focus of this study was on the high-frequency FM sweep-selective region of the pallid bat A1 (Razak and Fuzessery, 2002). This region is likely to be involved in echolocation behavior. The FM sweep-selective region contains neurons tuned between 25–70 kHz and is located rostral and medial to the lower frequency neurons (tuning 5–35 kHz) that are noise-selective (Razak and Fuzessery, 2002, 2006). The FM sweep-selective neurons respond better to downward sweeps than to noise or upward sweeps with energy in the same spectral band. Using tones, noise, upward, and downward sweeps as search stimuli, neurons with characteristic frequency (CF) >25 kHz, and with stronger response to downward FM sweeps than noise and upward FM sweeps were isolated. The following response properties were then determined:

### Excitatory frequency tuning curve

Pure tones (25–70 kHz, 5 ms duration, 1 ms rise/fall times, 1 Hz repetition rate) were used to determine the CF and MT for tones. CF was defined as the frequency that elicited action potentials to at least five successive stimulus repetitions at the lowest intensity. The intensity was then increased in 5 or 10 dB steps to record the frequency-intensity combinations that produced excitatory responses (tuning curve). A 1 kHz resolution was used to determine excitatory tuning curves. Because, the excitatory tuning curves in the echolocation region of the pallid bat are typically broader than 5 kHz between 10–30 dB above threshold, this resolution was deemed sufficient to characterize the tuning curve (Razak and Fuzessery, 2007).

### Two tone inhibition over time tuning curves

**Figure 1:**
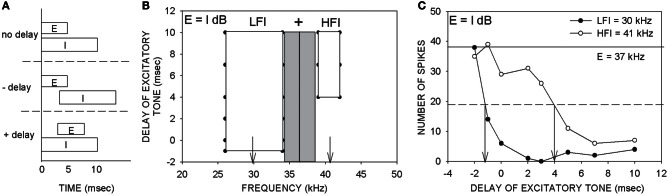
**Description of the two-tone inhibition method. (A)** An excitatory (E) tone at CF was paired with a putative inhibitory (I) tone with varying delays between the two tones. Positive delays indicate that the excitatory tone was delayed with respect to the inhibitory tone. Negative delays indicate earlier onset of the excitatory tone. **(B)** An example two-tone inhibition plot obtained with two tones of equal intensity (E = I dB). The gray rectangle indicates the excitatory tuning curve at the tested intensity. The vertical line indicates the CF used as the excitatory tone. The frequency of the putative inhibitory tone was varied from 25–70 kHz to determine the delay-frequency combinations that caused at least 80% reduction of response to the CF tone presented alone. The white rectangles represent the frequency-delay combinations that produced 80% inhibition. This neuron showed both low- and high-frequency inhibition (LFI, HFI). The vertical arrows indicate the LFI and HFI tones that were used to generate the plot shown in **(C)**. **(C)** An example two-tone inhibition plot in which response magnitudes at different delays between the tones were quantified. The intensities of the two tones were the same (E = I dB). The excitatory tone used was 37 kHz. The ‘number of spikes’ on the ordinate is in response to 20 repetitions of each stimulus. The solid horizontal line is the control response (response to CF alone). The dashed horizontal line is at 50% of control response. The LFI tone (30 kHz) produces at least 50% inhibition at a delay ~ −1 ms. The HFI tone (41 kHz) produces 50% inhibition only when the excitatory tone was delayed at least 4 ms. These data indicate that the LFI was relatively fast compared to HFI. The main goal of the present study was to obtain plots as shown in **(C)**, but by varying the relative intensities between the two tones and quantifying how arrival times of LFI and HFI change.

To determine the arrival time of inhibition, a two tone inhibition over time (TTI) method was used (Calford and Semple, 1995; Brosch and Schreiner, 1997; Gordon and O'Neill, 1998; Fuzessery et al., 2006; Razak and Fuzessery, 2006). Two tones were presented with different delays between them (Figure 1A). The frequency of one tone was at the CF (excitatory tone) and was presented at an intensity of 10–20 dB above threshold and duration of 5–10 ms. The second tone was presented at the same intensity and duration of 5–10 ms. The frequency of the second tone was varied between 25–70 kHz and its onset time was varied with respect to that of the excitatory tone. The inhibitory sideband on the low-frequency side is ~10 kHz wide (Razak and Fuzessery, 2006). On the high-frequency side, the bandwidth of inhibition is ~2–4 kHz. Therefore, a resolution of 0.5 kHz was used to search for the high-frequency sideband, and a resolution of 2 kHz was used for the low-frequency sideband.

The delay-frequency combinations of the two tones that resulted in inhibition of response to the excitatory tone for at least four out of five (80% inhibition) consecutive presentations served to map out the spectrum of inhibitory frequencies (Figure 1B, white rectangles). On the ordinate of the TTI plot in Figure 1B, negative delays denote that the onset of the excitatory tone occurred before that of the inhibitory tone. Positive delays indicate that the onset of the excitatory tone occurred after that of the inhibitory tone. The example neuron in Figure 1 exhibited both LFI and HFI. The timing of LFI and HFI was characterized in more detail (Figure 1C) by choosing an inhibitory tone at the center of the inhibitory sidebands (downward arrows in Figure 1B) and pairing it with the CF tone at various delays. In the description of TTI plots and data below, “control” response indicates response of the neuron to the excitatory tone presented alone. “Arrival time of inhibition” refers to the smallest delay between the two tones at which the response of the neuron was reduced by 50% of response to the control. It is important to note that arrival time of inhibition is a measure of when inhibition arrives at a neuron relative to excitation. In the example neuron shown in Figure 1C, the arrival times of LFI and HFI were −1 ms and +4 ms, respectively. Negative arrival times mean inhibition occurred even when the inhibitory tone was delayed relative to the excitatory tone. Therefore, negative arrival times are interpreted as fast arriving inhibition. Positive arrival times mean inhibition occurred only when the inhibitory tone was advanced relative to excitatory tone. Positive arrival times are interpreted as slow inhibition. The example in Figure 1 is typical of the pallid bat auditory cortex with slow HFI and fast LFI (Razak and Fuzessery, 2006).

### TTI at different relative intensities

In previous studies of the pallid bat auditory system, the TTI curves were obtained with the two tones at the same intensity as described above (e.g., Figure 1). These studies suggested that the differences in arrival time between LFI and HFI is a form of asymmetry that explain direction and rate selectivity for downward sweeps in the pallid bat auditory cortex and IC (Fuzessery et al., 2006; Razak and Fuzessery, 2006). However, it has been suggested that timing differences between the high- and low-frequency sidebands may be less important in shaping direction selectivity compared to how relative timing and magnitude of excitatory and inhibitory conductance interact with each other (Gittelman et al., 2009; Gittelman and Pollak, 2011). Modulation of magnitude of inhibitory/excitatory conductance may generate timing differences relevant to FM sweep selectivity. One way to test this hypothesis using extracellular recordings is by characterizing temporal interactions between the excitatory and inhibitory inputs change when the relative intensities of the two tones are varied.

Therefore, TTI tuning curves were determined at different relative intensities between the excitatory and the inhibitory tones. The excitatory tone was presented with an intensity 10–20 dB above threshold. The inhibitory tone was presented at different delays and intensities relative to the excitatory tone. The intensity of the inhibitory tone varied from 20 dB below to 20 dB above the excitatory tone intensity in steps of 5 dB. “Relative threshold of inhibition” refers to the lowest intensity of the inhibitory tone relative to the excitatory tone at which the neuron was inhibited by 50% of control response. Because the intensity of the inhibitory tone was changed in 5 dB steps, the resolution of the threshold of inhibition measure is 5 dB. Arrival time of both LFI and HFI was determined at different intensity combinations.

## Results

**Figure 2:**
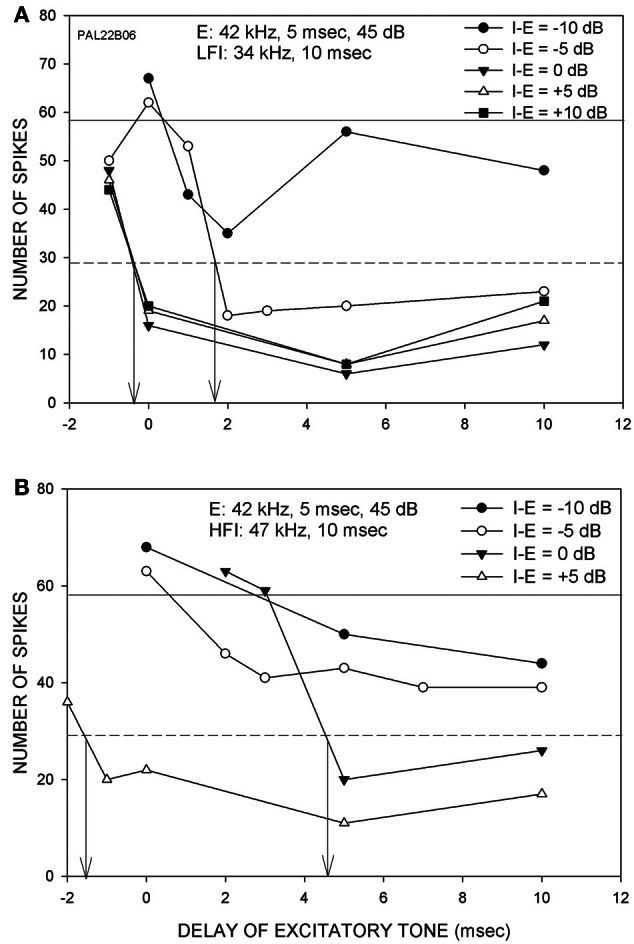
**Responses from a neuron (CF = 42 kHz) showing the effects of changing the intensity of inhibitory tone relative to the excitatory tone. (A)** The excitatory tone (E) was set at CF. The LFI was centered at 34 kHz. The intensity of E was fixed while the intensity of LFI tone was varied from 10 dB below (I-E = −10 dB) to 10 dB above (I-E = +10 dB) the E tone intensity. Solid horizontal line indicates response to E alone (control response). The dashed line marks 50% of control response. The vertical arrows mark the arrival time of LFI defined as the delay at which two-tone response decreased to 50% of control response. **(B)** In the same neuron, the effect of changing HFI intensity was also measured. For both LFI and HFI, the arrival time becomes faster with increasing relative intensity of inhibitory tone.

The goal of this study was to characterize changes in arrival time of inhibition when the intensities of the excitatory and inhibitory tones were changed relative to each other. This was accomplished in 33 FM sweep-selective neurons with CF between 30 and 51 kHz. In 15/33 neurons, stimulus was presented using the free-field speaker. In the remaining neurons, contralateral ear stimulation was used with a funnel inserted in the ear. Because no differences were found due to the method employed, the data are presented together. Figure 2 shows a neuron in which arrival times of both LFI and HFI were determined at multiple relative intensities. The CF (42 kHz) was used as the excitatory tone. The LFI tone used was 34 kHz (Figure 2A), and the HFI tone was 47 kHz (Figure 2B). When the LFI tone was presented at an intensity 10 dB lower than the excitatory tone (I-E = −10 dB), the response of the neuron decreased relative to the control response, but did not meet the 50% criterion to determine arrival time. When the intensity of the LFI tone was increased by 5 dB, while maintaining the excitatory tone intensity (I-E = −5 dB), the response of the neuron decreased below 50% of control response when the delay was ~1.5 ms. This intensity difference (I-E = −5 dB) was noted as the relative threshold of inhibition. At equal tone intensities, LFI arrival time was −0.5 ms. There was no reduction in the arrival time with a further increase in intensity of the inhibitory tone indicating a saturation of change in LFI arrival time. For the HFI, inhibition that met the 50% criterion was seen only when the two tones were of the same intensity (I-E = 0 dB), indicating a higher relative threshold for HFI than LFI. With a further 5 dB increase in intensity of HFI (I-E = 5 dB), the arrival time decreased to −1.5 ms. Indeed, at I-E = 5 dB, the arrival time of HFI was slightly faster than the arrival time of LFI. This example neuron lends support to the hypothesis the threshold of LFI was lower than the threshold of HFI, and that the arrival times of both LFI and HFI became faster with increasing relative intensity of inhibitory tone to a point of reduced temporal asymmetry.

**Figure 3:**
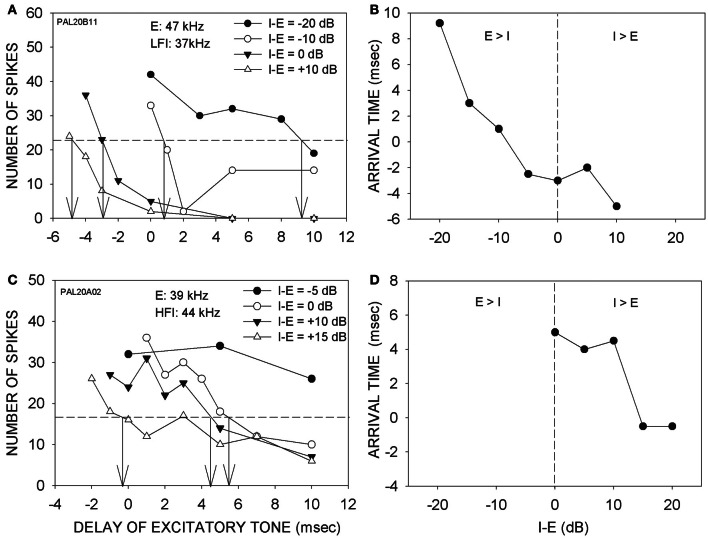
**(A)** Effects of changing relative intensity of inhibitory tone on LFI arrival time. Notations are as in Figure 3. **(B)** The panel shows the intensity-arrival time relationship for the neuron in **(A)**. Vertical dashed line indicates equal intensity of the two tones. **(C)** Effects of changing relative intensity of inhibitory tone on HFI arrival time. **(D)** The intensity-arrival time relationship for the neuron in **(B)**.

Figure 3 shows two additional neurons in which arrival times progressively decreased with increasing intensity of the inhibitory tone. In the neuron shown in Figure 3A, 50% inhibition was seen even when the LFI tone was 20 dB lower in intensity than the excitatory tone. With further increase in the inhibitory tone intensity, the arrival time of LFI decreased Figure 3B depicts the relationship between relative intensity and arrival time of LFI. For the neuron shown in Figures 3C,D, the HFI tone produced 50% inhibition only when the intensity of the two tones was equal. With further increase in the intensity of HFI, the arrival time decreased (Figures 3C,D).

**Figure 4:**
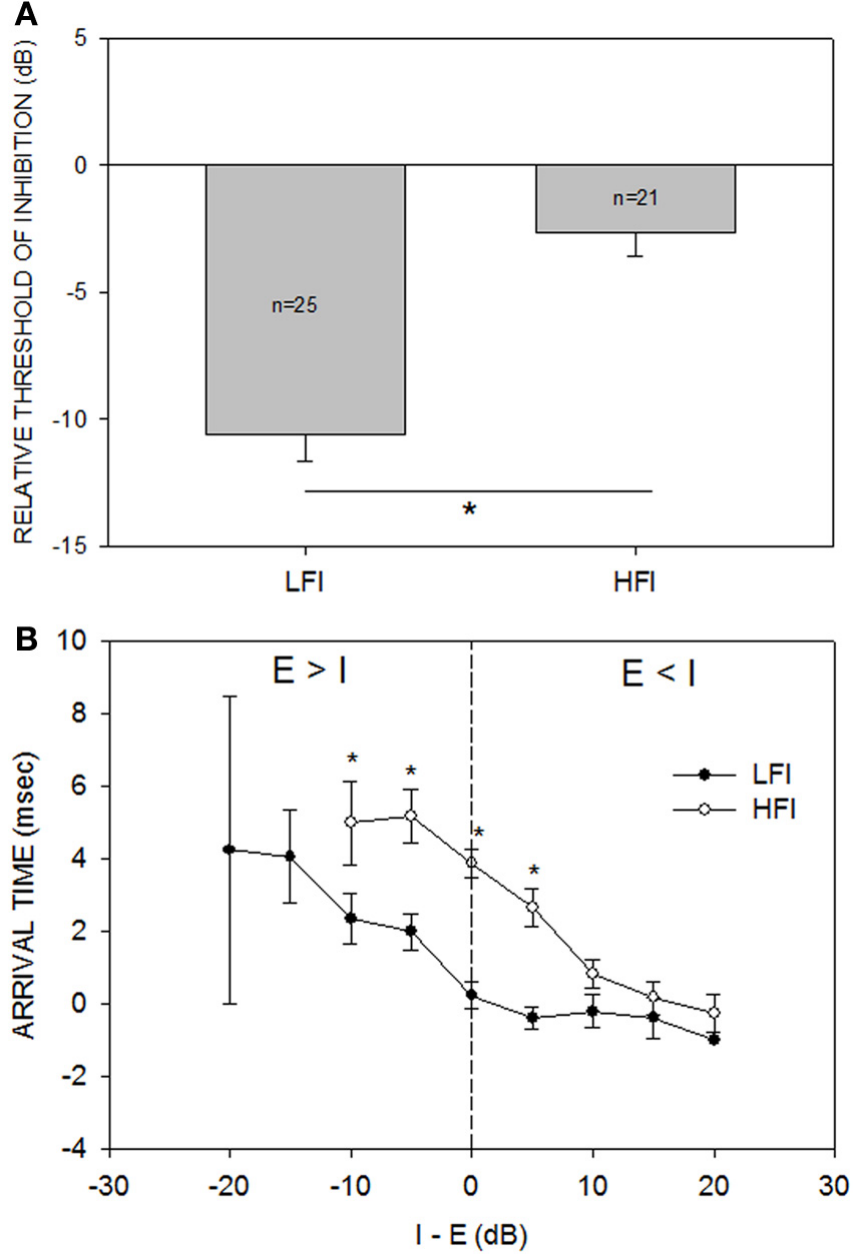
**(A)** The mean (±s.e.) relative threshold of inhibition of LFI was lower than that of HFI. Relative threshold of inhibition was defined as the smallest intensity difference between the inhibitory and excitatory tone that results in the neural response decreasing by 50% of response to excitatory tone alone (criterion inhibition). When tested with LFI tone, criterion inhibition was observed, on average, even when the inhibitory tone was 10 dB less intense than the excitatory tone. When tested with HFI, the two tones had to be of similar intensities, on average, to cause criterion inhibition. ^*^p < 0.001. **(B)** The mean (±s.e.) arrival time of LFI and HFI decreased as the intensity of the inhibitory tone was increased relative to the excitatory tone. “I-E” on the abscissa indicates the intensity difference between the inhibitory and excitatory tone. “Arrival time” on the ordinate indicates the shortest delay at which response to the two tones decreased to 50% of response to excitatory tone alone. The vertical dashed line marks the equal intensity point. There are no data points for HFI at −15 and −20 dB because HFI was not apparent when inhibitory tone was 15 or 20 dB less intense than the excitatory tone. The s.e.m for LFI at the −20 dB point is large because only two neurons showed criterion inhibition at this intensity difference.

Across the population (*n* = 33, Figure 4A), the relative threshold of HFI was significantly higher than that of LFI (Mann-Whitney Rank Sum Test, *p* < 0.001). On average, the HFI tone had to be similar in intensity to the excitatory tone to produce the criterion level inhibition. On average, LFI tone intensity produced criterion level inhibition even when its intensity was 10 dB lower than the excitatory tone. Figure 4B shows the population data for change in LFI and HFI arrival time with increasing intensity of the inhibitory tone. In general, the arrival time of HFI was slower than the arrival time of LFI. A two-way Anova and Tukey post-hoc pairwise tests comparing LFI and HFI arrival time showed significant differences (*p* < 0.05) at I-E values of −10, −5, 0, and +5 dB (asterisks in Figure 4B). However, this temporal asymmetry decreased as the intensity of the inhibitory tone was increased (arrival times not different at +10, +15, and +20 dB, Tukey post-hoc pairwise comparison, *p* > 0.05). When the two tones were of the same intensity, the arrival time of LFI was ~0 ms and the arrival time of HFI was ~4 ms, confirming previously published temporal asymmetries when the tones were of equal intensity (Razak and Fuzessery, 2006).

## Discussion

**Figure 5:**
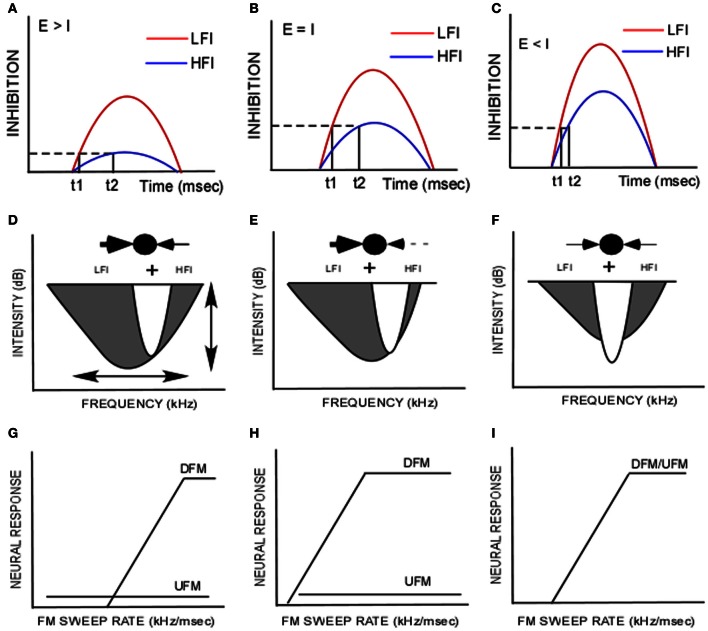
**(A–C)** The difference in arrival time of low-frequency inhibition (LFI, t1, red) and high-frequency inhibition (HFI, t2, blue) is related to the differences in threshold and amplitude of inhibitory input. The dashed line indicates a criterion level of inhibition. The solid vertical lines indicate the time at which the set level of inhibition is reached. **(A)** When the excitatory tone (E) is of greater intensity than the inhibitory tone (I), it is hypothesized that the HFI does not reach criterion level but LFI does, indicating a lower threshold for LFI than HFI. **(B)** When E = I, it is hypothesized that both LFI and HFI grow in strength, but LFI is stronger. It reaches the criterion inhibition sooner than HFI creating the temporal asymmetry relevant to FM sweep rate and direction selectivity as shown in Razak and Fuzessery (2006). **(C)** When E < I, both LFI and HFI grow in amplitude to a point of saturation resulting in reduced temporal asymmetry. **(D,E)** Schematic of inhibitory and excitatory components of the frequency receptive field. In the “ball and arrows” drawing, LFI is shown to the left and the HFI is shown to the right. **(G–I)** Idealized FM rate selectivity functions for upward and downward sweeps (UFM, DFM) for neurons with corresponding receptive fields shown in **(D–F)**. These schematics illustrate how species-specific FM sweep selectivity may be obtained by modifying two properties in a network, “synaptic strength” and “symmetry.” **(D)** A schematic of the strong asymmetry favoring the LFI in the pallid bat auditory cortex. This model illustrates the data from this paper that most neurons receive lower threshold LFI than HFI. The broader bandwidth of LFI is based on data in Razak and Fuzessery (2006). This gives rise to strong responses to downward sweeps in a rate-selective manner and weak/no response to upward sweeps. The vertical arrow in **(D)** indicates that relative synaptic strengths of HFI and excitatory input can be modified to obtain the tuning curve in **(E)**. The neuron is still strongly direction selective **(H)**, but is less selective for sweep rate compared to the neuron in **(G)**. The horizontal arrow in **(D)** indicates that the symmetry of LFI and HFI can be varied relative to the excitatory inputs. For example, if LFI and HFI are symmetrical in strength, bandwidth and timing, similar responses to upward and downward FM sweeps in a rate selective manner will result **(I)**.

The receptive field of auditory neurons includes both excitatory and inhibitory components (Arthur et al., 1971; Calford and Semple, 1995; Brosch and Schreiner, 1997; Gordon and O'Neill, 1998; Sutter et al., 1999; Faure et al., 2003; Wehr and Zador, 2003; Razak and Fuzessery, 2006; Wu et al., 2008; Sadagopan and Wang, 2010). The main goal of this study was to characterize the intensity dependence of temporal interactions between excitatory and inhibitory frequencies in auditory cortical neurons tuned in the echolocation range in the pallid bat. There were two main findings in this study (schematized in Figures 5A–D). First, the relative threshold of inhibition was lower for LFI than HFI (Figures 4A, 5A,D). On average, a LFI tone produced criterion inhibition even when its intensity was ~8–10 dB lower than the excitatory tone. A HFI tone produced criterion inhibition only when its intensity was the same or higher than the excitatory tone. Second, the arrival time of both LFI and HFI decreased when the intensity of the inhibitory tones was progressively increased relative to the excitatory tone (Figures 4B, 5A–C). With further increase in inhibitory tone intensity, the LFI and HFI arrival times reach a saturation level such that the temporal asymmetry decreases (Figures 4B, 5C). Thus, whether temporal asymmetry in sideband inhibition is present or not depends on the intensity relationship between the excitatory and inhibitory tones.

The echolocation call selective region of the pallid bat auditory cortex thus appears to be organized such that each neuron receives a stronger and lower threshold LFI input compared to the HFI input (Figure 5D). When tested with FM sweeps in which the LFI, HFI and excitatory frequencies have the same intensity, the evoked LFI will be stronger than the HFI. This difference in strength will translate into a difference in arrival time such that LFI arrives early while HFI has a longer latency compared to excitatory input. The temporal properties of inhibition are involved in shaping both FM rate and direction selectivity (Razak and Fuzessery, 2006; Razak, 2012). The early LFI will reduce responses to upward sweeps of any sweep rate while the slow HFI only reduces responses to downward sweeps with slow rates. This mechanism enhances direction selectivity while preserving responses to the fast downward sweeps used in echolocation.

These data suggest that the information that individual neurons provide is dynamic and depends on the echolocation context. During natural echolocation behaviors, the sweep selectivity of individual neurons will depend on the intensity distribution of various spectral components in the echolocation call. The intensity distribution of frequencies in the echo will, in turn, depend on many factors including the intensities in the call, directionality of call and ear, degree of environmental attenuation of different frequencies, and the distance to reflecting targets. For example, other factors being equal, a closer target may reflect a call with stronger energy in a neuron's HFI compared to a target that is further away. This will cause the neuron to be more selective for the faster sweep rates present in the echolocation call and selectively enhance responses to an echo relative to other sounds when the target is close-by. The other sounds include slower sweeps (e.g., communication calls) of either direction that overlap in spectrum (Brown, 1976; Kanwal et al., 1994; Bohn et al., 2008, 2009). As downward FM sweeps are commonly used signals to echolocate, a similar model may explain sweep direction and rate selectivity across FM bats. Low frequencies are attenuated less than high frequencies by the environment making it likely that low frequencies have relatively higher intensities than the high frequencies in returning echoes. The frequency-dependent environmental attenuation will add to the threshold differences between LFI and HFI noted here to generate strong downward sweep response bias. These neural and environmental factors may combine to partly explain the preponderance of downward sweep echolocation call usage by FM bats. In gleaners such as the pallid bat that depend on passive hearing for prey localization, the enhanced sweep selectivity provided by the model proposed here may also act to physiologically enhance the segregation of the parallel pathways used for echolocation and passive hearing (Barber et al., 2003; Razak et al., 2007). In fact, the larger percentage of direction selective neurons in the pallid bat auditory system compared even to other bats suggests that the strong LFI favoring asymmetry (stronger, faster, and lower threshold LFI) may serve pathway segregation more than FM processing.

### Mechanisms for species-specific FM sweep direction/rate selectivity

The pallid bat auditory system contains neurons with asymmetry that favors the LFI (Figure 5D). Manipulation (ontogenetically and phylogenetically) of two parameters in the model (Figure 5D) can lead to differences in FM sweep rate and direction selectivity. The first is *relative synaptic strength* of excitatory and inhibitory inputs. This can be thought of as variations along the ordinate of the tuning curve. The second is *asymmetry*, defined as the differences in properties between high- and low-frequency sidebands. This can be thought of as variations along the abscissa of the tuning curve. For example, if synaptic strength of LFI is stronger than excitatory inputs, then LFI will prevent responses to upward sweeps at any sweep rate (Figures 5D,E,G,H). The response of the neuron to downward sweeps will be sweep rate selective. The fastest rate that elicits a response will be determined by the difference in the strength of excitatory and HFI inputs. For example, a neuron with weak HFI will still generate rate selective response to downward sweeps, but the neuron will respond to a broader range of rates (Figures 5E,H). Likewise, changing the symmetry relationships between the inhibitory and excitatory components along the abscissa of Figure 5D will result in different levels of direction selectivity. Species in which direction selectivity is poor, neurons are predicted to have symmetric inhibition around the tuning curve (schematized in Figures 5F,I). Asymmetry favoring HFI will result in upward selectivity. A thorough characterization of spectral, temporal and intensity relationships between excitatory and inhibitory components of the receptive field is therefore required to understand the contribution of sideband inhibition to neural selectivity for FM sweeps.

### Mechanisms underlying intensity-latency relationship of inhibitory input

The two-tone inhibition paradigm has been used to study the interactions between excitatory and inhibitory components at different levels of the auditory pathway and across species. Most of these studies focused on the frequency-time (Calford and Semple, 1995; Brosch and Schreiner, 1997; Gordon and O'Neill, 1998) or frequency-intensity (Sutter et al., 1999) interactions, with few studies emphasizing intensity-time relationships (Arthur et al., 1971; Scholl et al., 2008; Sadagopan and Wang, 2010). In the rat auditory cortex, Scholl et al. (2008) tested the effect of changing relative levels of the two tones and found that the interactions were mostly suppressive, and did not shift to facilitation with intensity. The pallid bat cortex data are consistent with this finding in that no evidence for intensity-dependent switch from inhibition to facilitation was found. Together with the present study, these data indicate that interactions between at least three sound parameters: frequency, intensity, and time need to be characterized to describe inhibition in the receptive field.

The arrival time of inhibition determined using the two-tone inhibition paradigm is a measure of latency of inhibitory input relative to the excitatory input. It is established that the latency of excitatory tone responses decrease with increasing intensities above the MT (Klug et al., 2000). Latency saturates at a minimum value at a supra-threshold intensity and typically shows little change with further increase in intensity. Data from the present study suggest that the latency of inhibitory input also undergo similar intensity-dependent changes. With increasing intensity of the inhibitory tone, the arrival time of inhibition decreases systematically up to a point of saturation. It should be noted that arrival time of inhibition relative to excitation was measured here. However, the intensity of the excitatory tone was fixed suggesting that the change in arrival time was specific to inhibitory input. Latency of excitatory response can also show a non-monotonic relationship with intensity such that the latency reaches a minimum and then increases again with increasing intensity [paradoxical latency shift, (Galazyuk and Feng, 2001)]. No evidence for such a non-monotonic relationship for the arrival time of inhibition with intensity was found in the pallid bat cortex.

Although multiple mechanisms contribute to FM sweep rate/direction selectivity, asymmetric sideband inhibition is the dominant mechanism in the auditory cortex of the pallid bat (Fuzessery et al., 2011). Similar findings in rodent, primate and carnivore auditory systems indicate that these mechanisms are general principles of spectrotemporal processing and not just adaptations in an auditory specialist (Shamma et al., 1993; Zhang et al., 2003; Sadagopan and Wang, 2010; Ye et al., 2010; Kuo and Wu, 2012; Trujillo et al., 2013). In the pallid bat, spectrotemporal properties of sideband inhibition predict FM rate and direction selectivity. Exclusion of sideband frequencies from the sweep reduces/eliminates selectivity. Iontophoresis of antagonists of inhibitory neurotransmitters reduces/eliminates selectivity by altering sideband inhibition. These studies suggested that the sequence of excitatory and inhibitory inputs arriving at a neuron in response to FM sweeps influences selectivity for sweep direction and rate. Based on *in vivo* whole cell recording and modeling of IC responses, Gittelman and Pollak (2011) suggested that timing differences *per se* may be less important in shaping direction selectivity compared to interactions between relative timing and magnitude of excitatory and inhibitory conductance. Modulation of magnitude of inhibitory/excitatory conductance may generate timing differences relevant to FM direction selectivity (Figures 5A–C). This hypothesis is supported by findings in the rat auditory cortex where the relative timing of excitatory and inhibitory inputs depends on sound intensity (Wu et al., 2006) and in cat cortex in which latency of hyperpolarization in layer three pyramidal neurons decreases with sound intensity (Ojima and Murakami, 2002). The intensity-arrival time relationships in the cortex (current study), as well as the changes in arrival time of inhibition with inhibitory neurotransmitter receptor antagonists (Razak and Fuzessery, 2009; Williams and Fuzessery, 2011) are consistent with a relationship between strength and timing of inhibition. Threshold and strength of inhibition may therefore be substrates for modulation of timing of sideband inhibition implicated in FM sweep selectivity.

The decrease in latency of inhibitory input with increasing tone intensity may reflect faster excitatory inputs onto inhibitory neurons. In the rat auditory cortex, inhibitory input latencies advanced faster compared to excitatory latency when tested with tones at increasing intensities (Wu et al., 2006). Thus, the latency-intensity relationship may be stronger at the excitatory input to inhibitory neurons compared to excitatory inputs to excitatory neurons. In an integrate and fire model, the time taken for the membrane potential to reach threshold is determined by the maximum amplitude and rising slope of the post-synaptic potential (Wu et al., 2006). If the rising slope is steeper with sound intensity for the inhibitory conductance compared to excitatory input, the integration time will also be shorter for inhibition manifesting as faster latencies. The excitatory thalamocortical inputs to inhibitory neurons in the cortex are stronger than the inputs to excitatory neurons (Cruikshank et al., 2007). Thalamocortical excitatory currents rise faster in inhibitory interneurons than in excitatory neurons providing a basis for the faster advance of inhibitory latencies with increasing intensities compared to excitatory inputs.

It is noteworthy that in the rat cortex, the neurons in which inhibitory latencies advanced faster than excitatory latencies were non-monotonically tuned for sound intensity (intensity tuned neurons). In the pallid bat auditory cortex, the vast majority of the neurons in the echolocation region are also intensity tuned (Measor and Razak, unpublished observations). Thus, the intensity-latency relationship shown in the present study may not only shape FM sweep rate/direction selectivity, but also lead to intensity tuning for the echolocation calls. This hypothesis is currently being investigated.

### Conflict of interest statement

The author declares that the research was conducted in the absence of any commercial or financial relationships that could be construed as a potential conflict of interest.
